# Dynamic Behavior of Ti/Ti Single-Lap Laminated Structure with a Large-Diameter Bolt-Based Electromagnetic Force: Numerical Simulation and Experimental Verification

**DOI:** 10.3390/ma18071473

**Published:** 2025-03-26

**Authors:** Duquan Zuo, Shaoqing Jin, Tianyu Xu, Minghao Zhang, Mengyang Cui, Haolin Ding, Yaoming Fu

**Affiliations:** 1 Sichuan Province Engineering Technology Research Center of General Aircraft Maintenance, Civil Aviation Flight University of China, 46, Nanchang Road, Guanghan 618307, China; 2 College of Aviation Engineering, Civil Aviation Flight University of China, 46, Nanchang Road, Guanghua, 618307, China; m17772640663@163.com (S.J.); xty@cafuc.edu.cn (T.X.); 15039175214@163.com (M.C.); 15939045831@163.com (H.D.); 3 School of Materials Science and Engineering, Northwestern Polytechnical University, 127 West Youyi Road, Xi’an 710072, China; zhang_minghao@mail.nwpu.edu.cn

**Keywords:** TC4 alloy, large-diameter bolt, interference-fit bolted joint, electromagnetic force, finite element analysis, dynamic mechanical behavior

## Abstract

Electromagnetic force installation is recognized as a viable solution for interference-fit issues in large-diameter bolts. However, the dynamic mechanical behavior of the joint during installation has not been fully clarified. This study investigated the dynamic mechanical behavior of large-diameter Ti/Ti interference-fit bolted joints during electromagnetic installation through numerical simulation and experimental validation. The simulation results indicate uniform deformation at the inlet of the bore wall under interference levels, with a maximum displacement variance of 21.1 μm^2^. Axial stress distribution exhibited higher uniformity at 1% and 1.5% interference-fit amounts, demonstrating the capability of the electromagnetic-driven installation technique to ensure high-quality assembly within a defined interference range. The inlet-end stress consistently exceeded the outlet-end stress, while excessive interference (>1%) induced localized plastic deformation at the upper/lower plate inlets due to material softening. The critical interference threshold of 1% was identified: elastic deformation dominated below 1%, transitioning to plastic deformation beyond this limit. Thus, 1% interference is optimal for a Φ9.98 mm TC4 laminated structure. Furthermore, simulation and experimental results showed strong agreement, with installation force errors below 3.71%, validating the reliability and accuracy of the model in predicting dynamic interference-fit behavior.

## 1. Introduction

Titanium alloy materials are characterized by high specific strength, excellent corrosion resistance, and high toughness, making them widely used in critical structures of aircraft, such as frames, beams, and strings [1,2]. Currently, the primary connection methods for aircraft structures include riveting, bolting, bonding, and welding [3,4]. Among these, bolting stands out as the main assembly method due to its ease of disassembly, high load-bearing capacity, and simple structure, particularly in load-bearing structures [5]. However, traditional bolting techniques, such as pneumatic riveting and pressure riveting, suffer from low loading rates, small interference-fit levels, and significant joint damage [6], which fail to meet the requirements for non-destructive installation of large-diameter bolts by large interference-fit amounts. Electromagnetic force loading technology, with its advantages of high loading rates, high impact energy, and single-step forming, has garnered significant attention in the field of mechanical connections [7,8,9]. More precisely, electromagnetic force loading is essentially the forming process of a single high-speed impact [10], which can effectively solve issues in traditional connection techniques, such as significant bore wall damage, uneven radial extrusion, and small interference-fit levels. This method facilitates the uniform installation of large-diameter bolts with high levels of interference.

Numerous scholars have conducted experimental studies on the bolted joint in metal structures. Li et al. [11] performed quasi-static installation tests on CFRP/Ti laminated thin plates with a thickness of 6 mm using Φ6 mm interference-fit bolts and observed that excessive interference-fit levels could lead to significant grooving and grinding cracks on the titanium alloy surface, resulting in plowing plastic deformation. Zuo [12], using electromagnetic force to install Φ6.35 mm bolts, found that installation resistance increased with the amount of interference fit, but dynamic installation effectively reduced this resistance. Furthermore, Wang et al. [13] conducted installation tests on interference-fit structures composed of Φ4.81 mm bolts and 12.6 mm thick aluminum plates using an electromagnetic riveting gun, and they observed that electromagnetic force enabled the installation of fasteners with large interference-fit, providing higher installation quality compared to traditional methods. Pham et al. [14] studied the curling phenomenon in thin plates with a thickness of less than 1.9 mm and found that curling not only altered the bolt connection behavior but also affected its shear strength. Additionally, Zheng et al. [15] investigated the load-bearing capacity of hybrid bonded bolted joints in multi-rivet CFRP/Ti laminated thin plates with a diameter of Φ5 mm, finding that failure load increased with overlap distance. In summary, the electromagnetic force loading technology has been demonstrated to exhibit superior performance in reducing the installation resistance during the interference-fit installation of small-diameter bolts and improving the installation quality of joints. However, reports on the dynamic installation of medium-thickness plates and large-diameter interference-fit bolts under electromagnetic force loading are scarce, and the mechanical behavior of dynamic installation of threaded joints with high interference-fit levels remains unclear. Therefore, numerical and experimental studies are essential to be conducted for understanding their mechanical properties and further improving the installation quality and load-bearing capacity of joint structures.

In consideration of the aforementioned discrepancies, the present study focuses on the TC4 laminated structure with a single-lap joint, utilizing the ABAQUS simulation platform to simulate the dynamic interference-fit installation process of the large-diameter bolt under electromagnetic loading. The analysis covers four aspects: deformation displacement of the bore wall, stress magnitude and distribution, variation in installation forces, and cross-sectional temperature evolution. The mechanical behavior of large-diameter bolts under high levels of interference with electromagnetic force is revealed. Furthermore, experimental comparisons were conducted to validate the simulation results for installation forces at 1% and 1.5% amounts of interference fit, verifying the reliability and accuracy of the established model. This study offers reference guidance for subsequent experimental alternatives and provides theoretical underpinning for revealing the deformation mechanisms during the dynamic installation of large-diameter bolts. The main contents of the paper are summarized as follows. Section 2 delineates the verification experimental procedures and the establishment process of the finite element model. Section 3 conducts a multi-angle simulation-based analysis on the performance evolution and reliability assessment of large-diameter bolted joints under electromagnetic force loading, encompassing deformation and stress analysis, installation force analysis, temperature analysis, and experimental validation. Finally, Section 4 synthesizes a refined conclusion of the aforementioned investigations.

## 2. Experimental Study and Numerical Simulation

### 2.1. Experimental Design

**Figure 1:**
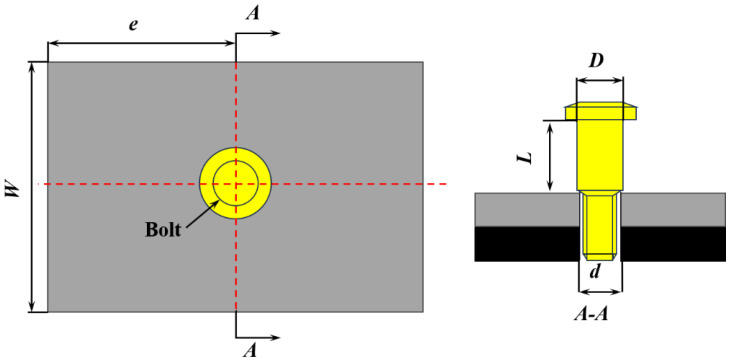
Geometry of the Ti/Ti interference-fit bolted joint.

Figure 1 illustrates the geometry of the Ti/Ti interference-fit bolted joint used by the specimen. The joint installation model was designed with the nominal width-to-diameter ratio (*W*/*d*) of 6 and the nominal edge distance ratio (*e*/*d*) of 4, where *W* is the specimen width in millimeters, *d* is the diameter of the titanium plate hole in millimeters, and *e* is the distance from the edge to the hole center in millimeters, with the edge effects on the experiment being neglected [16]. The TC4 titanium alloy plate has a thickness of 8 mm, and the high-lock bolt is made of TC4 with a diameter of Φ9.98 mm. Within the allowable engineering range [17], dynamic installation tests were conducted on bolted joints with relative interference-fit quantities (*α*) of 1% and 1.5%. The calculation of *α* is shown in Equation (1) [18],

(1) $$\alpha =\frac{D-d}{d}\times 100\%$$

where *α* represents the interference-fit quantity, *D* is the bolt diameter in millimeters, and the corresponding bore diameters to 1% and 1.5% amounts of interference fit are Φ9.88 mm and Φ9.83 mm, respectively.

**Figure 2:**
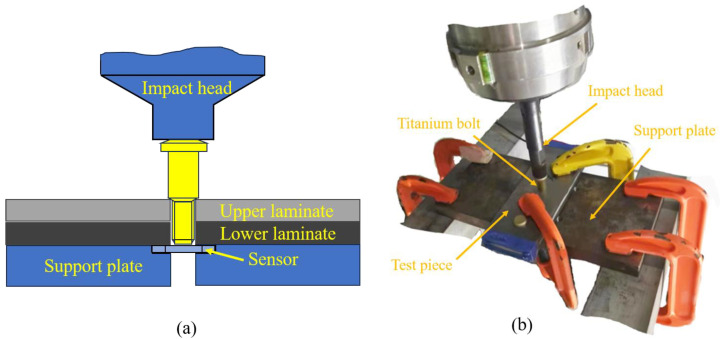
Dynamic installation test for the interference-fit bolted joint: (**a**) schematic diagram and (**b**) experimental process.

The EMD2000 electromagnetic loading system (developed by Northwestern Polytechnical University, Xi’an, China) was utilized to perform dynamic installation of the interference-fit bolted joints. Figure 2 illustrates the dynamic installation test for the interference-fit bolted joint. Initially, a clamping device secured the titanium plate to the support plate. The support plate was equipped with a through hole and groove for bolt installation and sensor embedding. During high-rate dynamic installation, the impact head charging voltage was set to 500 V to ensure effective installation of bolt fasteners within 1–5 ms, regardless of the values of interference fit [19]. The installation force was measured using a pressure sensor.

### 2.2. Model Establishment

**Figure 3:**
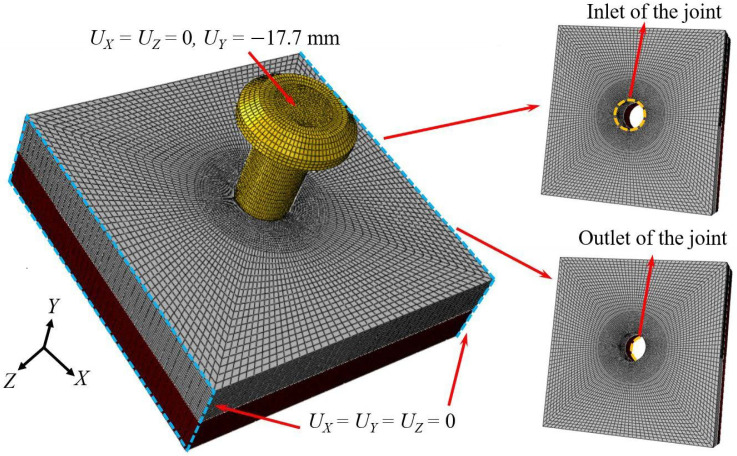
Simplification of the FE model used as the Ti/Ti interference-fit bolted joint.

The dynamic installation process of bolted joints under electromagnetic force loading was analyzed through FE modeling implemented in the ABAQUS 2022 simulation platform for the aforementioned experiment. The study focused on the deformation displacement of the bore wall at the joint, stress magnitude and distribution, axial installation force, and cross-sectional temperature. To comprehensively evaluate the feasibility and reliability of large-diameter bolts and high interference-fit installation, additional models with interference-fit amounts of 0.5% (corresponding to a diameter of Φ9.93 mm) and 2% (corresponding to a diameter of Φ9.78 mm) were introduced. Figure 3 illustrates a simplification of the FE model used as the Ti/Ti interference-fit bolted joint, where 3D 8-node reduced-integration thermal coupled continuum elements (C3D8RT) were utilized to simulate the changes in the wall of the bore. The Ti plate was meshed into 16 layers along the thickness direction. To ensure accuracy and efficiency in the simulation, the mesh was refined around the bore region, and the screw structure of the bolt fastener was neglected.

**Figure 4:**
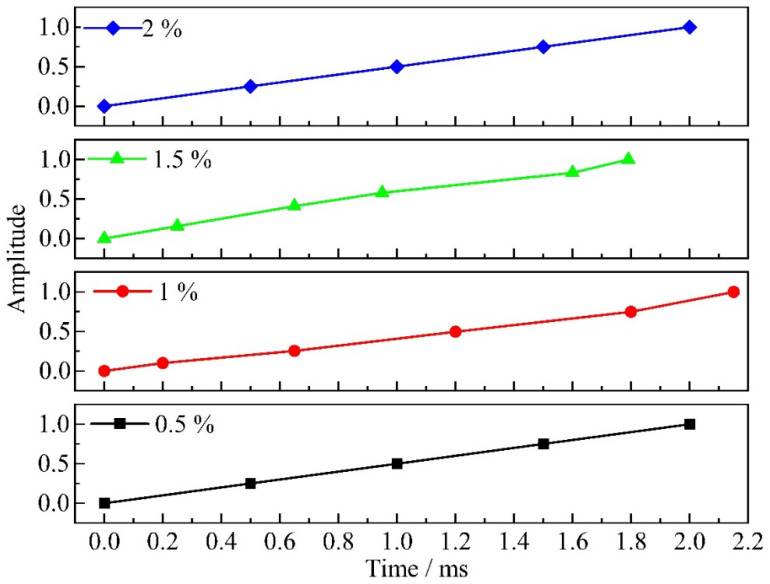
Relationship between bolt amplitude and time under different interference-fit levels.

The boundary conditions during the interference-fit installation process are shown in Figure 3, where the left and right end faces of the joint structure (indicated by blue rectangular frames in Figure 3) were fully fixed. To enhance the convergence of the simulation, a displacement load of 17.7 mm was applied to the bolt along the negative *Y*-axis based on actual experimental data, and the time-dependent load was controlled using amplitude functions (see Figure 4). This loading method effectively replicates the variable acceleration impact experienced for the bolt during actual dynamic interference-fit installation driven by electromagnetic force. The analysis step times for dynamic installation under 1% and 1.5% amounts of interference fit were defined as 2.15 ms and 1.79 ms, respectively, while a step time of 2 ms, derived from extensive electromagnetic force installation tests, was used for other interference-fit values [19].

The damage evolution of interference-fit bolted joints under electromagnetic impact loading was numerically characterized in the simulation using the Johnson–Cook constitutive model [20], which is an equation as follows:

(2) $$\sigma =(A+B\varepsilon^{n})(1+C\ln\frac{\dot{\varepsilon }}{{\dot{\varepsilon }}_{0}})[1-(\frac{T-T_{room}}{T_{melt}-T_{room}}{)}^{m}]$$

where *σ* is the equivalent stress (MPa), *ε* is the equivalent plastic strain, *έ* and *έ*_0_ are the strain rate and reference strain rate (s^−1^), respectively, *T* is the temperature (°C), *T_melt_* is the material melting point (°C), and *T_room_* is the room temperature (°C). The parameters *A*, *B*, *n*, *m*, and *C* represent experimental fitting parameters, with *A* as the initial yield stress (MPa), *B* as the strain hardening modulus (MPa), *n* as the strain hardening exponent, *m* as the thermal softening exponent, and *C* as the strain rate sensitivity. The Johnson–Cook constitutive parameters and corresponding material properties are listed in Table 1.

**Figure 5:**
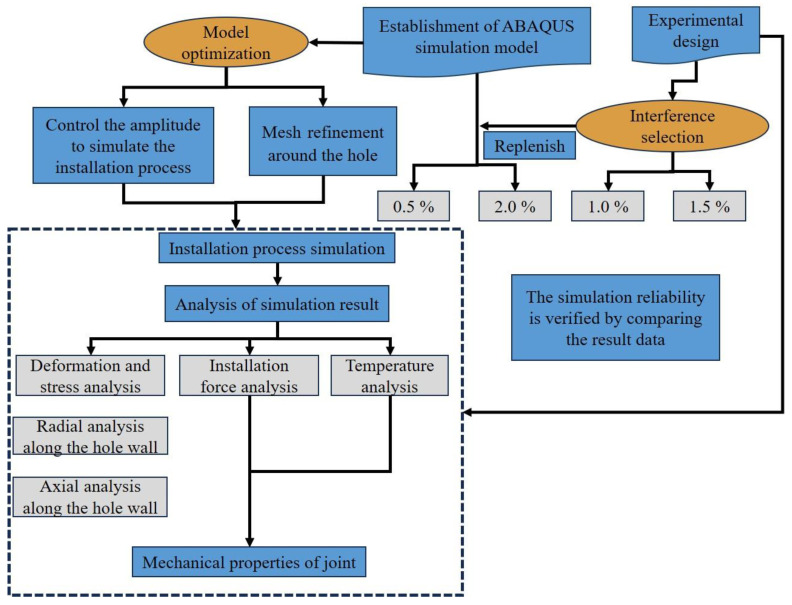
Workflow for investigating the dynamic mechanical performance of the joints.

In summary, this study conducts a comprehensive multi-angle analysis of the dynamic installation performance of large-diameter bolt interference-fit joints under electromagnetic force loading through a combined approach of simulation and experimentation, with the research workflow illustrated in Figure 5.

## 3. Simulation Results and Analysis

### 3.1. Deformation and Stress Analysis

#### 3.1.1. Radial Deformation and Stress of Bore Wall

The extent of damage at the inlet and outlet regions of the titanium plate hole was found to exhibit significant correlations with deformation and stress distribution in the bore wall of the bolted joint [21]. Enhanced stress concentrations were observed at the inlet region, attributed to the combined effects of large hole dimensions and initial bolt-bore wall contact under electromagnetic loading, which intensified localized mechanical responses [22]. Therefore, the radial displacements at the inlet were chosen for analysis, as shown in Figure 4.

**Figure 6:**
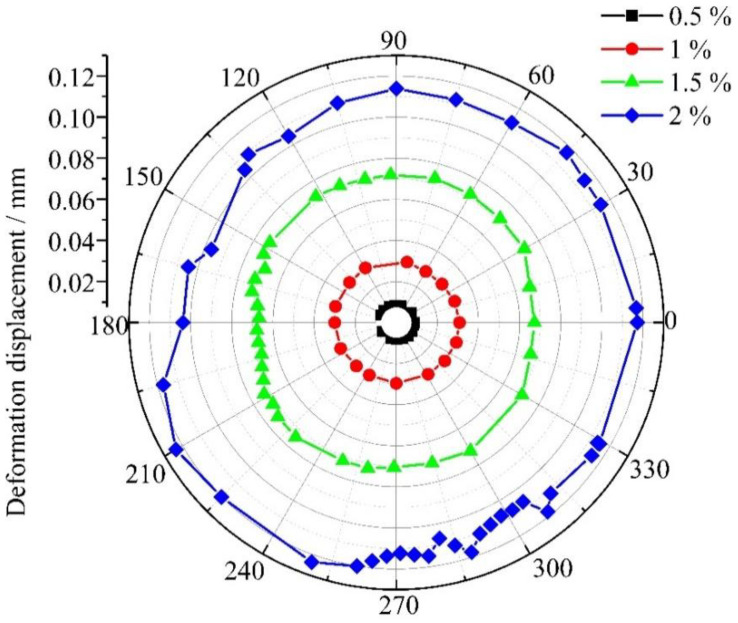
Radial displacement distribution at the inlet aperture of joints under varying interference-fit levels.

As indicated in Figure 6, the radial displacements of the bore wall increase progressively as the interference-fit levels range from 0.5% to 2%, with the range of displacement fluctuations expanding accordingly. To quantify the deformation quality more precisely, variance was introduced to describe the dispersion of radial displacement. As shown in Figure 6, 12 radial displacement values at 30° intervals were used to compute the variance, defined by the following Equation (3) [23],

(3) $$S^{2}=\frac{1}{n}\sum_{i=1}^{n}{\left(x_{i}-x_{0}\right)}^{2},(i=1,2\cdots 12)$$

where *S*^2^ is the sample variance in µm^2^, *x_i_* represents the radial deformation displacement at different angles in µm, *x*_0_ is the mean radial deformation displacement in µm, and *n* is the number of angles. Table 2 presents the variance of radial deformation displacements on the surface of the bore wall.

From Figure 6 and Table 2, it is evident that the interference-fit level increases, and the maximum variance of radial deformation displacement at the inlet of the joint also increases (from 0.005 µm^2^ to 21.1 µm^2^). The maximum deformation variance is only 21.1 µm^2^, approximating 0, indicating a low degree of deformation displacement dispersion. Therefore, a relatively uniform distribution of radial displacement deformation at the inlet was observed under electromagnetic force loading across varying interference levels.

**Figure 7:**
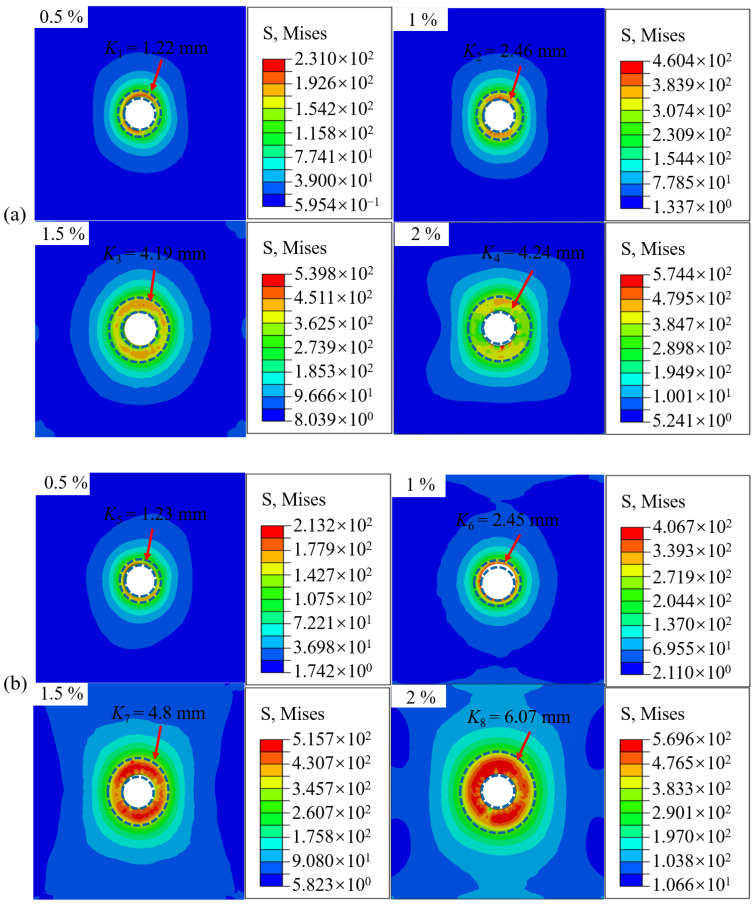
Radial stress contours of the bore wall on joints without bolt under different interference-fit levels: (**a**) inlet and (**b**) outlet.

**Figure 8:**
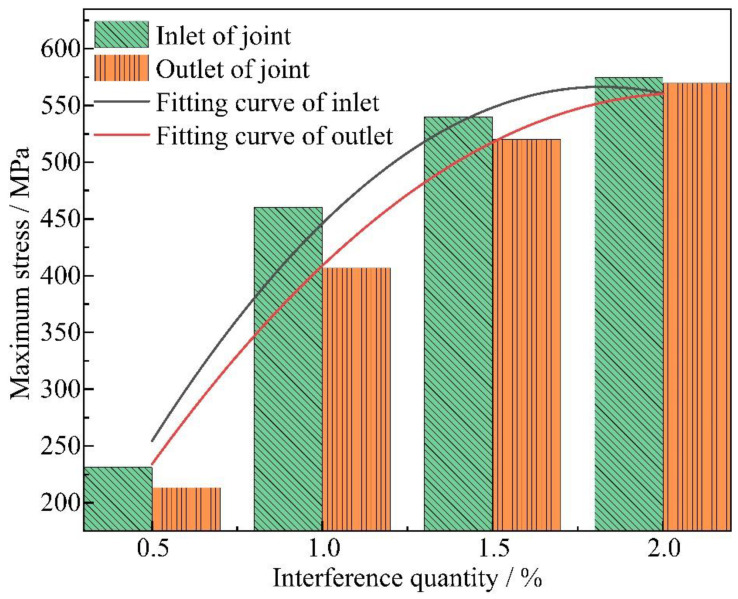
Maximum stress variation trends stress at the inlet and outlet regions of the joint under varying interference-fit levels.

The interference-fit bolted joint is subjected to stress concentration and non-uniform loading in the bore wall region, particularly at the inlet and outlet zones, under high-speed electromagnetic impact loading, attributable to the enlarged bore geometry [24] and elevated strain rates. Radial stress contour diagrams at the inlet and outlet regions of the bore wall across interference-fit levels ranging from 0.5% to 2% are displayed in Figure 7, whereas maximum stress variation curves at these critical regions are depicted in Figure 8.

As observed in Figure 7, the stress ring region at the inlet of joints expands from *K*_1_ (1.22 mm) to *K*_4_ (4.24 mm), with the degree of stress variation (in the red region) intensifying as the interference-fit level increases (becoming wider and redder). Similarly, the stress ring range at the outlet end expands from *K*_5_ (1.23 mm) to *K*_8_ (6.07 mm), with *K_j_* (*j* = 5~8) showing consistent stress variation with *K_i_* (*i* = 1~4). Further analysis reveals that the stress ring at the outlet is slightly higher than at the inlet, and the degree of stress variation is more severe, particularly under the large interference-fit quantity of 2%. This is due to the increased installation resistance and lack of rigid support at the outlet as the bolt insertion depth and interference-fit level increase.

To elucidate the relationship between the interference quantity and the maximum stresses at the import and export ends, a quadratic fitting method was employed in this study to derive the trend line of maximum stress variation at the import end, as illustrated in Figure 8. The fitted formulas for stress variation at both the import and export ends are presented in Equations (4) and (5), with coefficients of determination R^2^ being 0.9922 and 0.9998, respectively.

(4) $$y=-194.8x^{2}+708.92x-69.5$$

(5) $$y=-143.8x^{2}+595.98x-48$$

where *x* is the interference quantity in % and *y* is the maximum stress in MPa. Further analysis revealed that with the interference-fit amount increasing from 0.5% to 2%, the maximum stress at the inlet and outlet of joint increases parabolically, with the slope of the curve gradually flattening. This phenomenon is due to the greater the interference-fit amounts, the larger the radial extrusion force as the contact area between the bolt and hole increases, which in turn raises the friction coefficient. Consequently, higher electromagnetic loading energy is required, naturally leading to greater stress at the inlet and outlet of joint. However, as the insertion depth increases, the axial contact area between the bolt and bore wall expands, and when the frictional heat accumulation becomes excessive, the material undergoes a softening effect [25]. This also explains why the maximum stress at the inlet of joint is generally higher than at the outlet.

#### 3.1.2. Axial Stress and Deformation of the Bore Wall

**Figure 9:**
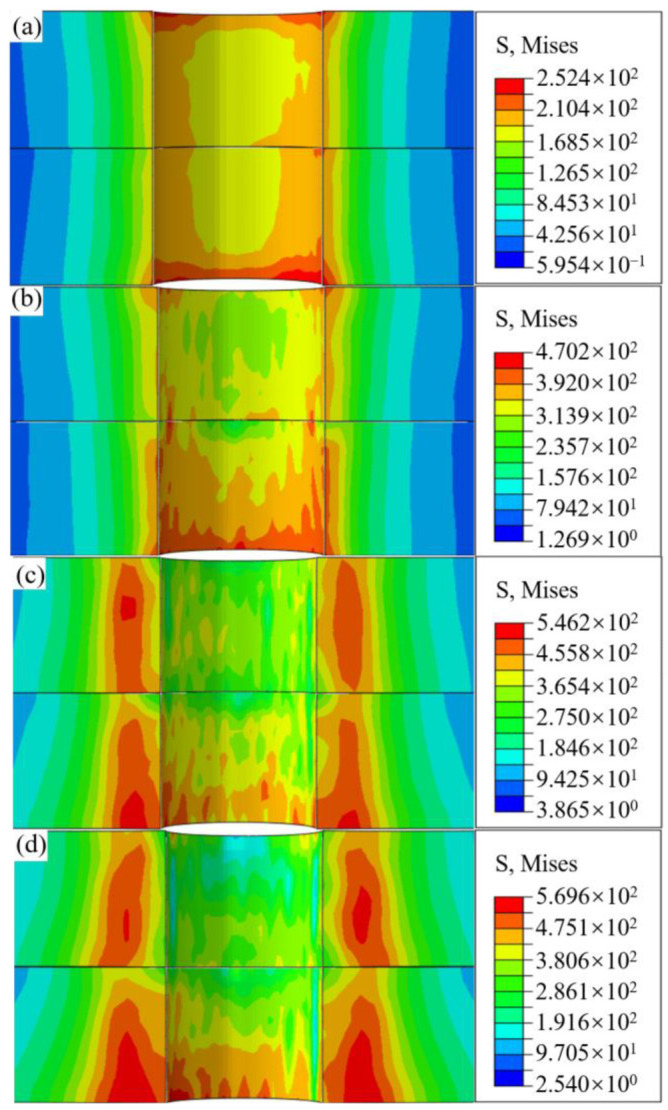
Axial stress contours of the bore wall on joints without bolts under interference-fit levels at (**a**) 0.5%, (**b**) 1%, (**c**) 1.5%, and (**d**) 2%.

The axial stress contour of the boltless bore wall is presented in Figure 9. Regardless of interference-fit levels, radially outward propagation of stress from the inlet of the upper plate bore wall is observed, with its magnitude progressively diminishing. As the bolt advances toward the upper plate outlet, circumferential stress distribution attains its peak range, particularly pronounced at interference-fit levels of 1.5% and 2%. Upon insertion into the lower plate, the stress distribution pattern remains almost identical to that in the upper plate. Additionally, it is evident that the stress ring distribution along the axial direction is more uniform for interference-fit amounts of 0.5% and 1%, demonstrating the high installation quality achieved by the dynamic installation technique with electromagnetic force for large-diameter bolted joints within a certain range of interference-fit.

Furthermore, Figure 9 shows that, regardless of interference-fit levels, the maximum stress region is located at the outlet of the bore wall. As the interference-fit amount increases from 0.5% to 2%, the radial range of the red stress region gradually expands. This indicates that excessive interference causes an accumulation of frictional energy, which is not dissipated in time, resulting in significant deformation heat at the outlet of the bore wall. Further analysis reveals that the incremental change in maximum stress decreases with increasing interference-fit amounts: 217.8 MPa (from 0.5% to 1%), 76 MPa (from 1% to 1.5%), and 23.4 MPa (from 1.5% to 2%). This is due to the transition of material from elastic to plastic deformation caused by deformation heat, leading to the softening effect [26]. This conclusion aligns with the results shown in Figure 7 and Figure 8. Therefore, it can be inferred that the softening effect is a significant factor in the stress reduction observed during high interference installation of the bolted joint.

**Figure 10:**
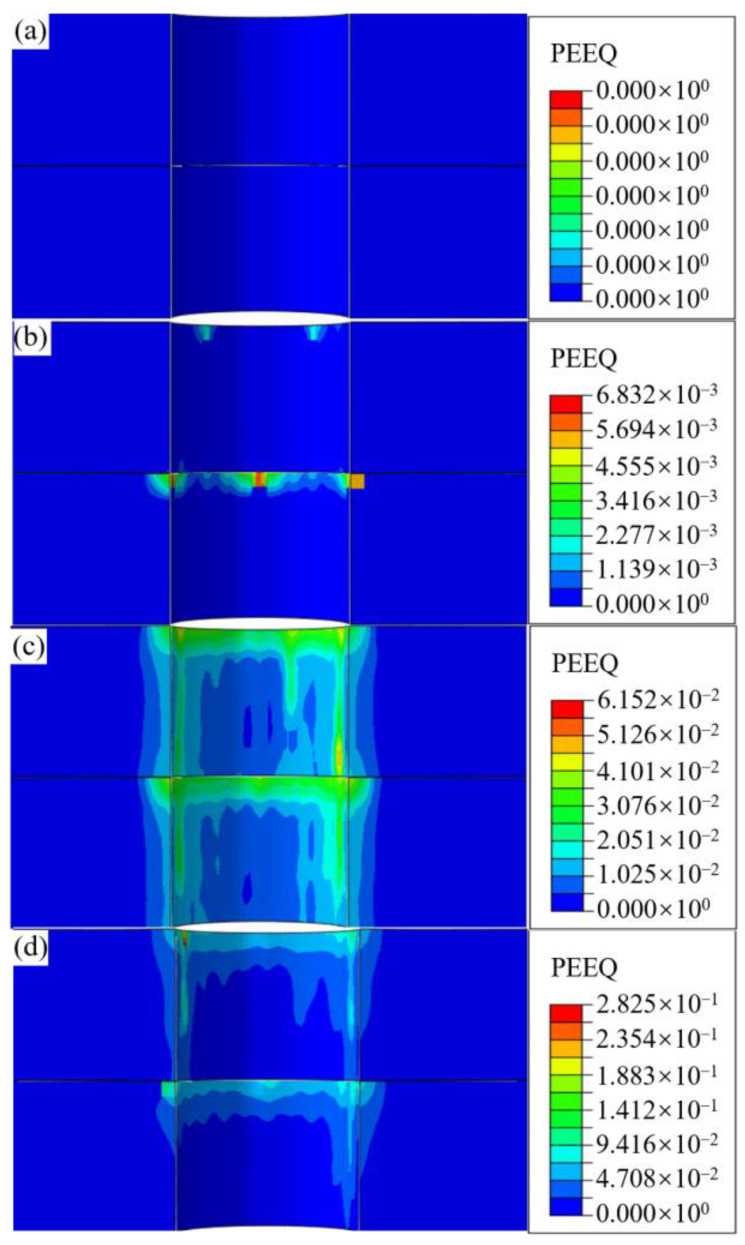
Equivalent plastic strain contours of the bore wall on joints without bolts along the axial direction at (**a**) 0.5%, (**b**) 1%, (**c**) 1.5%, and (**d**) 2%.

Equivalent plastic strain is a critical indicator of the deformation mechanism in the bore wall material [27]. Three distinct zones are defined based on the correlation between interference-fit levels and bore wall stress, which are elastic, elastoplastic, and plastic influence zones [28,29]. In the elastic influence zone, where stress remains below the material yield strength, small interference-fit levels are observed, and only elastic or minor plastic deformation is induced. Conversely, under relatively large interference-fit levels within the plastic influence zone, significant plastic deformation is generated in the bore wall material. The elastoplastic influence zone serves as the transition region, wherein material stress attains the yield strength, and a gradual shift from elastic to plastic deformation dominance occurs. Figure 10 shows the axial equivalent plastic strain of the bore wall without bolts under different interference levels.

The axial distribution characteristics of equivalent plastic strain along the bore wall under varying interference-fit conditions are quantitatively depicted in Figure 10. It is observed that when the interference-fit amounts do not exceed 0.5%, the bore wall material does not undergo plastic deformation. Conversely, as the interference increases, the plastic strain begins to appear in the bore wall material, with strain increments of 6.8 × 10^−3^ (from 0.5% to 1%), 546.9 × 10^−3^ (from 1% to 1.5%), and 2209.8 × 10^−3^ (from 1.5% to 2%). This indicates that the deformation severity increases with a higher interference fit, illustrating a transition from the elastic influence zone to the plastic influence zone under high interference-fit installation. In Figure 10b, when the interference levels reach 1%, the bore wall exhibits minor plastic deformation, with a plastic strain of only 6.8 × 10^−3^, suggesting that the installation resistance is approaching the yield strength threshold of the material. This indicates that the critical interference-fit percentage for the designed bolted joint is around 1%. When the interference amounts are below 1%, the bore wall primarily undergoes elastic or minor plastic deformation; above this threshold, significant plastic deformation occurs, mainly concentrated at the inlet of the upper and lower plates due to energy accumulation from the insertion depth and the large bore boundary effect [30] at the inlet of the bore wall.

### 3.2. Installation Force Analysis

**Figure 11:**
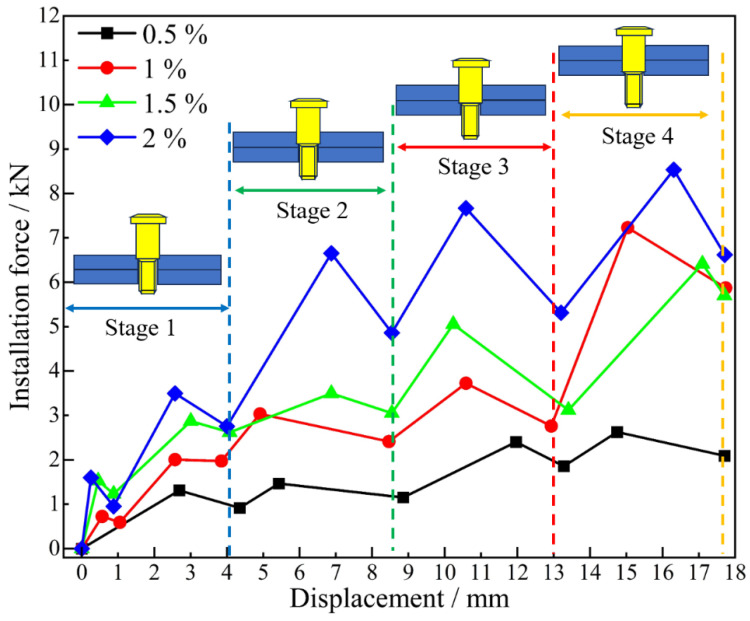
Relation curves of installation force and displacement at varying interference-fit levels.

The electromagnetic force generated during bolt dynamic installation is predominantly converted into dual components: axial frictional forces and radial extrusion forces applied to the bore wall material [31]. As evidenced by the force–displacement characteristics presented in Figure 11, the deformation mechanisms and stress states undergone by the joint during the installation phase can be systematically identified. For enhanced characterization of installation force evolution in large-diameter bolt dynamic interference installation processes, installation phase segmentation into four distinct stages has been implemented: Stage 1: Bolt is inserted halfway into the upper plate (corresponding to the plate thickness of 4 mm); Stage 2: Bolt reaches the outlet of the upper plate (corresponding to the plate thickness of 8 mm); Stage 3: Bolt is inserted halfway into the lower plate (corresponding to the plate thickness of 4 mm); and Stage 4: Bolt reaches the outlet of the lower plate (corresponding to a plate thickness of 8 mm).

In Stage 1, as the large-diameter bolt is inserted into the upper plate, elastic deformation occurs at the inlet, and the installation resistance causes the force to increase linearly. As the insertion depth increases, the frictional contact area between the bolt and the bore wall enlarges, causing significant heat accumulation and elastic relaxation (i.e., minor plastic deformation) of the bore wall material, leading to a slight instantaneous drop in installation force. As the bolt continues to the half-thickness position of the plate, the installation force curve first rises and then falls. When the bolt progresses through Stages 2, 3, and 4, the trend in the installation force mirrors the latter part of Stage 1, exhibiting a stepped, fluctuating increase [32]. This phenomenon is due to the interplay between the elastic and plastic zones of the bore wall material during interference-fit installation, causing the material stress to oscillate around the yield strength.

### 3.3. Temperature Analysis

**Figure 12:**
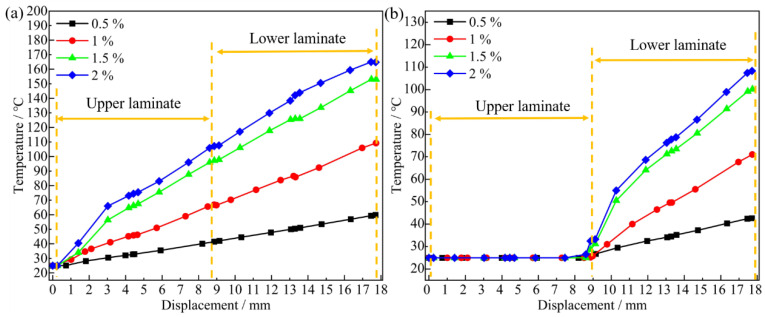
Temperature-displacement curves at different inlet cross-sections of bore wall for joints: (**a**) upper plate and (**b**) lower plate.

The temperature variations caused by energy accumulation are key to understanding the deformation of the bore wall during dynamic installation [33,34]. To explore the relationship between deformation and temperature, the temperatures at the inlet cross-sections of the upper and lower plates under different interference-fit amounts were extracted. Figure 12 shows the temperature variation curves at the inlet of bore wall cross-sections. For analysis, the temperature variation curves are divided into upper and lower plate sections. Figure 12a indicates that when the values of interference-fit are ≤ 1%, the inlet temperature increases linearly. Conversely, for interference > 1%, the temperature curve initially rises rapidly and then increases approximately linearly. This phenomenon indicates that as the interference-fit amount increases, the installation resistance grows due to a higher friction coefficient, causing a rapid temperature rise, especially for interference amounts > 1%. The contact area between the bolt and the bore wall becomes too large, the installation resistance surges with the radial extrusion force, and the accumulated energy converts to heat quickly, leading to an initial steep rise in temperature. Subsequently, due to heat conduction, the temperature diffuses, and the rate of increase slows.

As seen in Figure 12b, when the bolt approaches the inlet of the lower plate, the temperature change slope begins to vary, exhibiting a high consistency with the upper plate inlet temperature curve. For interference > 1%, the temperature change slope at both the upper and lower plates at the inlet significantly increases, indicating that the bore wall material transitions between elastic and minor plastic deformations with increased bolt insertion depth. The critical interference-fit percentage required for bore wall stress to attain yield strength is determined to be approximately 1%, which is corroborated by the analytical findings presented in Figure 10.

### 3.4. Experimental Validation

**Figure 13:**
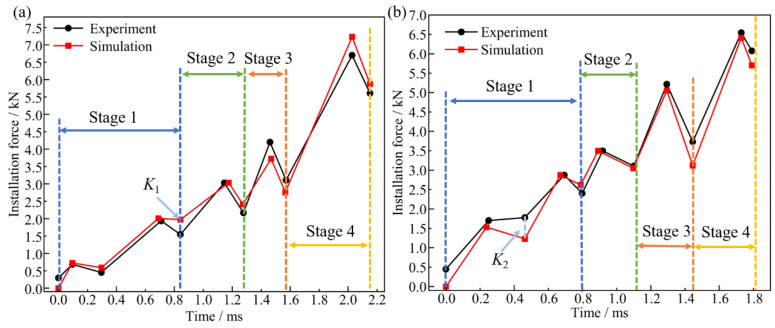
Comparison curves of experimental and simulation results for installation force at various interference-fit levels: (**a**) 1% and (**b**) 1.5%.

Electromagnetic-driven dynamic installation processes of Ti/Ti interference-fit bolted joints were systematically executed under controlled pulse parameters, and force–time curves were used to validate the simulation results. The comparison between simulation and experimental results is shown in Figure 13. The maximum installation force error between simulation and experimental data does not exceed 3.71%, which is below the engineering requirement of 5%. The close agreement between the simulation and experimental curves indicates a high level of reliability for the established simulation model.

The higher installation force errors at points *K*_1_ (27.75%) and *K*_2_ (30.51%) may result from the impact nature of electromagnetic loading, causing slight vibrations in the interacting laminate structure due to high-speed bolt driving. Further analysis shows that for interference-fit amounts of 1% and 1.5%, the installation time in Stage 1 is 0.84 ms and 0.8 ms, respectively, which are 1.45 to 2.80 times and 2.29 to 2.67 times longer than in other stages. This suggests that, during the initial installation stage, the bolt accumulates a significant amount of energy to overcome the installation resistance of the bore wall.

## 4. Conclusions

To predict the stress and deformation of the bore wall at the joint without a bolt under electromagnetic loading, this study performed numerical simulations and experimental validations of the dynamic installation process for large-diameter interference joints. The deformation displacement, stress–strain distribution, installation force variation, and cross-sectional temperature evolution were compared and analyzed for different interference-fit levels. The main conclusions are as follows:

1. The variance in radial deformation displacement at the inlet of joints with different interference-fit levels is less than 21.1 μm^2^, approximating 0, indicating uniform radial deformation of the bore wall. Additionally, at interference-fit levels of 1% and 1.5%, the axial stress distribution on the bore wall is more uniform compared to other interference amounts, demonstrating that dynamic installation under electromagnetic loading provides high installation quality for large-diameter bolts within a specific range of interference.
2. The stress at the inlet of the bore wall is generally higher than at the outlet. For large interference-fit installation, plastic deformation of the bore wall is primarily concentrated at the inlet of the upper and lower plates. Furthermore, the maximum stress increment in the bore wall (ranging from 217.8 MPa to 23.4 MPa) gradually decreases, and installation force is slightly dropped during the initial stage. This indicates that the softening effect induced by large interference fit is a critical factor affecting the dynamic installation process.
3. The critical interference-fit threshold at which bore wall stress states attain yield strength equivalence is quantitatively established at approximately 1%. Below this critical threshold, the material response is predominantly characterized by elastic deformation mechanisms, whereas beyond this magnitude, plastic deformation modes become the governing mechanical behavior. This elastoplastic transition behavior fundamentally defines the optimal installation interference magnitude as 1% for joint integrity preservation. Furthermore, a high correlation is identified between bore wall inlet deformation characteristics and cross-sectional temperature fluctuation patterns.
4. At interference-fit amounts of 1% and 1.5%, the installation time in Stage 1 is 1.45 to 2.8 times and 2.29 to 2.67 times longer compared to other stages, respectively. This indicates that a significant amount of energy is required to overcome the installation resistance of the bore wall during the initial stage. The high degree of fit between experimental and simulation results, with errors not exceeding 3.71% and below the engineering requirement of 5%, confirms the reliability of the simulation model and the accuracy of its results.

This study, conducted through simulation and experimental investigations, concludes that electromagnetic force loading technology exhibits superior performance in the dynamic interference-fit installation of medium-thick plates and large-diameter bolts, thereby providing valuable references for future advancements in the aviation industry. Furthermore, in light of the limitations identified in the current research, further investigation into the mechanisms governing installation is deemed imperative.

## Figures and Tables

**Table 1:** Johnson–Cook model parameters assignment and performance parameters.

Material	*A*/MPa	*B*/MPa	*n*	*m*	*C*	*T_melt_*/°C	*T_room_*/°C
TC4	862.4	901.7	0.341	0.767	0.018	1652	25
Material	Young’s modulus /GPa		Poisson’s ratio		Density /g∙cm^−3^		Specific heat/J∙(kg∙K)^−1^
TC4	114		0.33		4.45		678

**Table 2:** Variance of radial deformation displacement at joint inlet under different interference-fit levels.

Interference Quantity/%	0.5	1	1.5	2
Variance/μm^2^	0.005	0.478	4.131	21.1

## Data Availability

The data presented in this study are available on request from the corresponding author due to project implementation constraints.
